# A Comparison of Warm-Up Effects on Maximal Aerobic Exercise Performance in Children

**DOI:** 10.3390/ijerph192114122

**Published:** 2022-10-29

**Authors:** Avery D. Faigenbaum, Jie Kang, Michael DiFiore, Caitlyn Finnerty, Andy Garcia, LeeAnn Cipriano, Jill A. Bush, Nicholas A. Ratamess

**Affiliations:** Department of Kinesiology and Health Sciences, The College of New Jersey, Ewing, NJ 08628, USA

**Keywords:** cardiopulmonary, dynamic warm-up, exercise test, heart rate, youth

## Abstract

The aim of this study was to compare the warm-up effects of treadmill walking (TW) with a dynamic (DY) bodyweight warm-up on maximal aerobic exercise performance in children. Sixteen children (10.9 ± 1.5 vrs) were tested for peak oxygen uptake (VO_2_ peak) on 2 nonconsecutive days following different 6 min warm-up protocols. TW consisted of walking on a motor-driven treadmill at 2.2 mph and 0% grade whereas the DY warm-up consisted of 9 body weight movements including dynamic stretches, lunges, and jumps. Maximal heart rate was significantly higher following DY than TW (193.9 ± 6.2 vs. 191.6 ± 6.1 bpm, respectively; *p* = 0.008). VO_2_ peak (54.8 ± 9.6 vs. 51.8 ± 8.7 mL/kg/min; *p* = 0.09), maximal minute ventilation (68.9 ± 14.8 vs. 64.9 ± 9.4 L/min; *p* = 0.27), maximal respiratory exchange ratio (1.12 ± 0.1 vs. 1.11 ± 0.1; *p* = 0.85) and total exercise time (614.0 ± 77.1 vs. 605 ± 95.0 s; *p* = 0.55) did not differ significantly between DY and TM warm-ups, respectively. These findings indicate that the design of the warm-up protocol can influence the heart rate response to maximal aerobic exercise and has a tendency to influence VO_2_ peak. A DY warm-up could be a viable alternative to a TW warm-up prior to maximal exercise testing in children.

## 1. Introduction

Cardiopulmonary exercise testing is a valid and reliable method for evaluating aerobic fitness and is commonly used by pediatric exercise scientists and clinicians to assess the functional capacity of children and adolescents [1,2]. Over the past few decades there has been an increase in pediatric exercise testing and the development of child-specific equipment and assessment technologies have improved the quality of the data obtained [1,2]. With growing evidence indicating that aerobic fitness is an important marker of health in youth, increased attention has focused on the role of pediatric exercise testing and the importance of standardized testing methodologies [3,4,5]. 

The measurement of maximal or peak oxygen uptake (VO_2_ peak) during pediatric cardiopulmonary exercise testing is considered the single best measure of aerobic fitness and can serve as a biomarker for the development of health outcomes [2,5,6]. Since only a minority of children satisfy the classic VO_2_ plateau criteria at maximal exercise, the term VO_2_ peak is often used when describing maximal aerobic exercise capacity in youth [4,7]. While there are different treadmill (TM) test protocols that can be used to determine VO_2_ peak in youth, exercise testing typically begins with an initial warm-up period followed by graded exercise with increasing loads [1]. A warm-up prior to an aerobic exercise test can help to prepare the body for the demands of subsequent exercise and may influence peak exercise performance [4]. Inbar and Bar-Or published a small but important study reporting that VO_2_ peak and maximal heart rate were significantly higher in 7- to 9- year old boys following an intermittent 15 min warm-up (30 s run at 60% VO_2_ peak, 30 s rest) compared with no warm-up [8]. Other pediatric researchers found that a general warm-up (low-intensity running and stretching) and an inspiratory muscle warm-up (performed with an inspiratory muscle training device) increased acceleration and sprint performance in 12- to 14-year old boys more than control conditions [9]. Most research examining running performance and maximal aerobic exercise capacity in children does not report warm-up procedures in detail. 

While there are no accepted warm-up protocols for pediatric exercise testing, general warm-up guidelines for a TM exercise test include 5 to 8 min of submaximal walking [4]. A general warm-up can increase body temperature, blood flow, heart rate and respiration rate [10,11]. A dynamic (DY) warm-up consists of a series of low-, moderate- and high-intensity movements such as stretching, lunging, jumping and multidirectional sprints that are designed to prepare participants for the demands of the upcoming activity [12,13]. The potential benefits of a DY warm-up include the aforementioned benefits of a general warm-up and an elevation in oxygen uptake kinetics (e.g., oxygen delivery and extraction), mobilization of joints (e.g., range of motion) and activation of the neuromuscular system (e.g., post activation potentiation) [10,13]. In addition, a DY warm-up can help to mentally prepare participants for subsequent exercise by establishing a desired tempo, facilitating readiness, and building self-confidence with task-relevant instructor feedback [10,12]. 

Previous research has found that it may be desirable for youth to perform a DY warm-up prior to the performance of activities that require a high power output (e.g., jumping and sprinting) [14,15,16]. The possibility that a DY warm-up could influence the responses to maximal aerobic exercise could have important implications for physical education teachers, youth coaches, clinicians, and pediatric researchers who administer field-based (e.g., Progressive Aerobic Cardiovascular Endurance Run) or laboratory-based (e.g., graded exercise test) cardiopulmonary assessments to children. Differences in pre-exercise test protocols may alter physiological processes and cognitive factors that may affect performance during cardiopulmonary exercise testing. Accordingly, the purpose of this study was to compare the warm-up effects of traditional TM walking with a DY bodyweight warm-up on the cardiopulmonary responses to maximal aerobic exercise in children. We hypothesized that a DY warm-up would elicit higher cardiopulmonary responses to maximal aerobic exercise than TM walking in children.

## 2. Materials and Methods

### 2.1. Participants 

Sixteen children (11 boys and 5 girls; mean ± SD age = 10.9 ± 1.5 yr; height = 142.8 ± 9.2 cm and body mass = 36.3 ± 6.7 kg) volunteered to participate in this study. Participants were active members of local sports teams (primarily basketball, lacrosse and soccer). Parents completed a modified physical activity readiness questionnaire to evaluate the health status of the participants and assess the safety for performing vigorous exercise. Exclusion criteria included the following: cardiopulmonary or metabolic disease; orthopedic limitation; or positive responses from parents to one or more of the questions pertaining to their child’s health. No participant was excluded from participation. All parents signed a parental permission form and all participants signed a child assent form and were informed of the benefits and risks of this investigation. The study was conducted according to the guidelines of the Declaration of Helsinki and approved by the Institutional Review Board at the College (2019-0214). 

### 2.2. Protocols

Peak Aerobic Capacity Testing. All participants reported to the Human Performance Laboratory on 2 occasions at the same time of day (within 7 days) at least 2 h postprandial for peak aerobic capacity testing. All tests were conducted by the same investigator with support from research assistants under controlled temperature conditions. Participants were asked to refrain from vigorous exercise (e.g., sports competition) for at least 24 h before each testing session. Participants were given standardized instructions before the start of the test. VO_2_ peak was assessed using the Fitkids treadmill test protocol and a metabolic system (MedGraphics ULTIMA Metabolic System, MedGraphics Corporation, St Paul, MN, USA). The system was calibrated prior to each test according to the manufacturer’s instructions. The Fitkids treadmill test is a valid and reproducible exercise test for children that consists of 90 s stages with incremental increases in speed and incline until volitional exhaustion [17].

Each participant was fitted with a child-size respiratory mask that was placed over the participants face, fastened, and carefully checked for proper sealing. Breath-by-breath VO_2_ data were obtained and VO_2_ peak was determined by recording the highest measure observed during the test. Values for minute ventilation (V_E_) and respiratory exchange ratio (RER) were recorded during the entire protocol. HR was monitored using a soft chest strap with a HR sensor (Model A300; Polar Electro Inc, Woodbury, NY, USA). Heart rate data were downloaded for analysis using a computer software program. HR peak was defined as the highest value achieved during the test. Criteria for accepting the exercise test as maximal included HR peak >180 bpm, a respiratory exchange ratio (RER) > 1.0, and signs of intense effort (unsteady gait, facial flushing, and clear unwillingness to continue despite verbal encouragement) [18]. Ratings of perceived exertion (RPE) were also monitored. Participants were asked to manually signal their RPE during the test on a visually presented scale with a numerical response range of 0–10 [19]. Prior to testing, height was measured to the nearest 0.1 cm using a wall-mounted stadiometer and body mass was measured to the nearest 0.5 kg using an electronic scale. For both measurements, participants wore light clothes and no shoes.

Warm-up Protocols. Participants performed different 6 min warm-up protocols in random order prior to peak aerobic capacity testing. The DY warm-up protocol consisted of 9 body weight exercises that included lower intensity and higher intensity movements (Table 1). In order to familiarize participants with the DY warm-up protocol, a research assistant demonstrated each DY exercise at the desired cadence and participants observed proper technique and movement speed prior to testing. A research assistant performed the DY warm-up protocol with each participant during testing procedures. Participants were asked to complete 5 repetitions (or 10 s) of each DY exercise (on each side when appropriate). Proper technique was reinforced with child-friendly coaching cues. All participants performed the same exercises in the same order. The DY warm-up protocol used in our study was designed to prepare children for maximal aerobic exercise by elevating oxygen uptake kinetics and neuromuscular potentiation without undue fatigue. The DY warm-up exercises were based on prior research examining the effects of different warm-up protocols on physical performance and our personal experiences designing youth fitness programs [14,20,21]. The TW warm-up protocol consisted of walking on a motor-driven treadmill at 2.2 mph and 0% grade. The TW protocol used in this study was designed to be a moderate-intensity walking protocol that is consistent with traditional warm-up recommendations prior to pediatric exercise testing [4,22]. Heart rate data were collected during both warm-up protocols. 

Participants performed a vertical jump test using the Vertec Jump Trainer (Sports Imports, Hillard, OH, USA) one minute after each warm-up protocol. The best jump of three trials was recorded. The purpose of the vertical jump test was to assess if either warm-up protocol influenced neuromuscular potentiation which may create an optimal environment for intense exercise [10,11]. Following completion of the vertical jump test, participants sat for 3 min while they were prepared for peak aerobic capacity testing.

### 2.3. Statistical Analysis

To determine the minimum sample size required, a priori power analysis was run by the software GPower (version 3.1.9) with a desired power level of 0.80, an alpha level of 0.05 and effect size calculated from previous studies [8,23]. Descriptive statistics (mean ± SD) were calculated for all dependent variables. A paired *t*-test was used to compare HR, VO_2_, VE, RER, RPE, exercise test time, and vertical jump between warm-up protocols. For all statistical tests, a probability level of *p* ≤ 0.05 denoted statistical significance. Effect size was calculated using Cohen’s d. Statistical analyses were conducted in SPSS (version 24; SPSS, Chicago, IL, USA).

## 3. Results

The peak cardiorespiratory responses to maximal aerobic exercise testing following the DY and TW warm-up protocols are outlined in Table 2. Paired sample t-tests revealed peak heart rate was significantly higher following DY than TW. A trend towards significance was observed for VO_2_ peak (*p* = 0.09). No significant differences between the DY and TW protocols were found for maximal V_E_, maximal RER, total exercise time, and maximal RPE (8.7 ± 0.7 and 8.7 ± 0.8, respectively; *p* = 0.85). There were no significant differences following DY and TW for vertical jump (36.1 ± 7.5 vs. 36.1 ± 7.3 cm; *p* = 0.91). 

The mean HR during the DY warm-up was significantly higher than the TW warm-up (126.7 ± 17.8 and 108.7 ±13.7 bpm, respectively, *p* = 0.0006). No significant differences were found for the vertical jump test following the DY and TW warm-up protocols (36.1 ± 7.5 and 36.1 ± 7.3 cm, respectively; *p* = 0.91). No significant order effects between test day 1 and test day 2 were observed for any peak cardiorespiratory variable. No adverse events occurred during or after any testing session.

## 4. Discussion

To address the lack of information about warm-up effects prior to maximal aerobic exercise testing in children, we compared the effects of two different warm-up protocols on the cardiorespiratory responses to maximal aerobic exercise performance in children. The main findings were that peak heart rate was significantly higher following a DY warm-up than a TW warm-up and that there was a tendency for a DY warm-up to improve VO_2_ peak in children. Similar responses were found for maximal V_E_, maximal RER, maximal RPE and total exercise time. Both warm-up protocols were well-tolerated by the participants. These findings indicate that a DY warm-up could be a viable alternative to a traditional TW warm-up prior to maximal treadmill exercise testing in children. These findings are in partial agreement with our initial hypothesis proposed.

Since most research examining aerobic exercise capacity in children does not report warm-up procedures in detail, it is difficult to compare our findings from the existing body of literature due to the paucity of evidence regarding warm-up effects on maximal aerobic exercise in youth. In our study maximal heart rate following the DY and TW protocols were 193.9 bpm and 191.6 bpm, respectively. These findings are consistent with results from an early study that found maximal heart rate in 7- to 9-year-old boys following a cycle test was 196 bpm after an intermittent running warm-up and 188 bpm with no warm-up [8]. In our study heart rate during aerobic capacity testing was significantly higher following the DY warm-up than the TW warm-up during and after stage 2 of the exercise test.

Our findings support the contention that a priming bout of pre-event dynamic movements may augment the cardiopulmonary responses to subsequent aerobic exercise [24,25,26]. The cause for the differences in exercise heart rate following different warm-up protocols is not entirely clear. The higher submaximal and maximal heart rates achieved during the exercise test following DY as compared to TM may be due, at least in part, to greater sympathetic nervous system stimulation consequent to the priming effects of the DY protocol [1,25]. In our study, mean heart rate during the DY and TW warm-up protocols increased from 112.4 bpm and 108.8 bpm, respectively, during minute 1 to 141.0 bpm and 110.2 bpm, respectively, during minute 6. Heart rate during the DY and TM protocols reached 73% and 57%, respectively, of peak heart rates achieved during maximal aerobic capacity testing. During the DY protocol, participants performed a series of learned body weight exercises at progressively higher intensities. It has been demonstrated that the cardiovascular responses during exercise are governed by both central, i.e., central commend, and peripheral, i.e., pressor reflex, mechanisms [27]. In this context, the relatively intense and more engaging nature our DY protocol could explain why the HR response was higher not only during the DY warm-up protocol but also at VO_2_ peak. The priming effects of higher intensity warm-up procedures on the cardiopulmonary responses to maximal exercise have been found to persist for 30 to 45 min [28].

In our study there were no significant differences between the DY and TW protocols for maximal V_E_, maximal RER, maximal RPE, and total exercise time. Values for maximal V_E_ and maximal RER were indicative of intense effort [1]. A trend towards significance was observed for VO_2_ peak. Participants in our study engaged regularly in sport activities and had an average VO_2_ peak of 54.8 mL/kg/min and 51.8 mL/kg/min following the DY and TW protocols, respectively. Although the difference in VO_2_ peak was not statistically significant, a ~5% difference in performance could have practical applications. For example, a 1% difference in aerobic capacity could influence the outcome of competitive distance running events. 

The priming effects of prior exercise have been found to alter oxygen uptake kinetics during subsequent exercise in adults [26,29,30], and it seems similar findings could occur in youth depending upon the intensity and duration of the warm-up protocol [24]. In support of this observation, during stage 1 of the exercise test oxygen uptake was significantly higher following the DY protocol than the TW protocol (data not shown). In an early study Gerbino and colleagues found that 6 min of high-intensity but not moderate intensity exercise increased VO_2_ kinetics during subsequent heavy exercise in adults [30]. Although speculative, a higher intensity dynamic warm-up may be needed to change metabolic function and improve aerobic exercise tolerance in children.

Well-designed warm-up protocols can prime the cardiorespiratory and neuromuscular systems for more vigorous exercise [10,31]. Proposed mechanisms that may influence subsequent high-intensity exercise include greater oxygen availability, differences in enzyme activation, increased muscle blood flow and improved motor unit recruitment patterns [10,31]. In our study, participants performed a vertical jump test 1 min following each warm-up protocol and there was no significant difference in jumping performance between trials. Since the DY warm-up did not elicit improvements in neuromuscular performance in our study, it appears other mechanisms influenced subsequent exercise performance. It is also possible that the intensity and duration or our DY protocol were not sufficient to facilitate neuromuscular potentiation.

The DY warm-up used in our investigation appeared to be a safe warm-up protocol without any adverse events, and possibly more engaging, enjoyable and less boring than TW. We observed good acceptability and tolerability by all participants to the DY warm-up which may be due to the child-friendly design of our DY protocol, individualized instruction and close monitoring. Due to the age of the participants and the relative intensity of selected DY exercises, a progressive protocol with a short recovery interval between exercises was arguably required to maintain safety, motivation and adherence. Although the affective response to the DY protocol was not explored in our investigation, we observed that our DY protocol was challenging and appealing for the participants as evidenced by 100% compliance with research instructions and testing protocols. 

Our study has strengths and limitations. This was one of the first studies to compare warm-up effects on maximal aerobic exercise performance in children. Cardiopulmonary capacity was assessed with direct measures of oxygen consumption and the testing order was randomized. Since the sample included active children the generalizability of the results and conclusions to other populations including those with illnesses or disabilities that alter movement or mechanical efficiency may be limited. There is the potential for biological maturity to confound the results because maturation was not assessed and therefore we were unable to determine if all participants were prepubertal. It is also important to consider the design of our DY protocol because the cardiopulmonary responses to pre-event dynamic exercise are dependent upon various factors including the intensity of muscle actions, the amount of muscle mass used, body position, warm-up duration and rest interval between the warm-up and the subsequent exercise test.

## 5. Conclusions

The present study demonstrated that the design of the warm-up protocol can influence the heart rate response to maximal aerobic exercise in children and has a tendency to influence VO_2_ peak. These findings indicate that differences in pre-test procedures may affect the accuracy of pediatric exercise testing and the consequent interpretation of exercise test data. While there are no standard warm-up procedures for pediatric exercise testing, our findings indicate that a DY warm-up could be a viable alternative to a TW warm-up. Considering the increased use of exercise testing in clinical and research settings, our findings along with high compliance to our study procedures provide support for continued innovation and research examining the warm-up effects of different protocols on maximal aerobic exercise performance in children.

## Figures and Tables

**Table 1:** Description of dynamic warm-up exercises.

1. Toe taps and heel taps Stand in staggered stance with hips forward and shoulders back; tap foot 5x then tap heel 5x. Repeat on other side	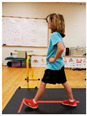 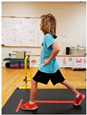
2. Split squats Stand in split leg position; lower hips until back knee is just above the floor; quickly stand back up and return to the starting position with rear heal down; repeat 5x then repeat on other side	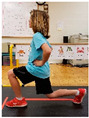
3. Plank with foot flutters Place forearms on floor with elbows above shoulders and body in straight line; flutter right and left foot to heal height of opposite foot 5x each	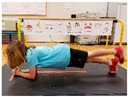
4. Hip bridge Lie on back with arms at side; quickly raise hips to create a straight line from knees to shoulders; return to starting position and repeat 5x	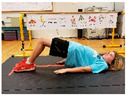
5. Trunk rotation Kneel with 1 hand on floor and 1 hand behind head with elbow pointing towards floor; rotate away from hand on floor with shoulder over shoulder; return to starting position; repeat 5x then repeat on other side	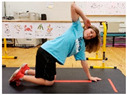
6. Side leg raise Lie on side with body in straight line and head on arm; lift leg off floor about 12 inches then return to starting position; repeat 5x then repeat on other side	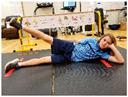
7. Snap down/snap up Stand with arms extended overhead; quickly snap arms down towards knees, pause briefly, then quickly return to starting position; repeat 5x	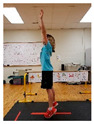 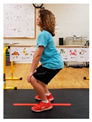
8. Line jump Stand behind line on the floor with feet hip-width apart. Quickly jump back and forth across the line for 10 sec. Repeat jumping side to side for 10 sec.	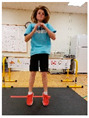 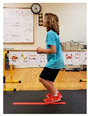
9. Squat jumps Stand with feet hip width apart; Quickly jump straight up and swing arms overhead; repeat 5x	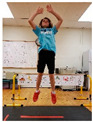

**Table 2:** Peak cardiometabolic responses during maximal aerobic capacity exercise testing following a dynamic warm-up (DY) and a treadmill walking warm-up (TW).

	DY	TW	*p*-Value	Effect Size (*d*)
HR, bpm	193.9 ± 6.2	191.6 ± 6.1	0.008	0.32
VO_2_, mL/kg/min	54.8 ± 9.6	51.8 ± 8.7	0.09	0.23
V_E_, L/min	68.9 ± 14.8	64.9 ± 9.4	0.27	0.31
RER	1.12 ± 0.1	1.11 ± 0.1	0.85	0.02
Exercise time, s	614.0 ± 77.1	605 ± 95.0	0.55	0.11

VO_2_, oxygen uptake; HR, heart rate; V_E_ minute ventilation; RER, respiratory exchange ratio.

## Data Availability

Data are available upon reasonable request to the corresponding author.
